# Functional Effects of Epilepsy Associated *KCNT1* Mutations Suggest Pathogenesis via Aberrant Inhibitory Neuronal Activity

**DOI:** 10.3390/ijms232315133

**Published:** 2022-12-01

**Authors:** Grigori Y. Rychkov, Zeeshan Shaukat, Chiao Xin Lim, Rashid Hussain, Ben J. Roberts, Claudia M. Bonardi, Guido Rubboli, Brandon F. Meaney, Robyn Whitney, Rikke S. Møller, Michael G. Ricos, Leanne M. Dibbens

**Affiliations:** 1 Clinical and Health Sciences, Australian Centre for Precision Health, University of South Australia, Adelaide, SA 5000, Australia; 2 School of Biomedicine, University of Adelaide, Adelaide, SA 5005, Australia; 3 South Australian Health and Medical Research Institute, Adelaide, SA 5005, Australia; 4 Clinical and Health Sciences, Health and Biomedical Innovation, University of South Australia, Adelaide, SA 5000, Australia; 5 Department of Woman’s and Child’s Health, Padua University Hospital, 35128 Padua, Italy; 6 The Danish Epilepsy Centre, 4293 Dianalund, Denmark; 7 Denmark Department of Clinical Medicine, Copenhagen University Hospital, 2200 Copenhagen, Denmark; 8 Division of Neurology, Department of Paediatrics, McMaster University, Hamilton, ON 8SL 4L8, Canada; 9 Department of Epilepsy Genetics and Personalized Treatment, Member of the ERN EpiCARE, The Danish Epilepsy Centre, 4293 Dianalund, Denmark; 10 Department of Regional Health Research, University of Southern Denmark, 5000 Odense, Denmark

**Keywords:** epilepsy, K^+^ channels, patch clamping, channelopathies, gain-of-function mutations

## Abstract

KCNT1 (K^+^ channel subfamily T member 1) is a sodium-activated potassium channel highly expressed in the nervous system which regulates neuronal excitability by contributing to the resting membrane potential and hyperpolarisation following a train of action potentials. Gain of function mutations in the *KCNT1* gene are the cause of neurological disorders associated with different forms of epilepsy. To gain insights into the underlying pathobiology we investigated the functional effects of 9 recently published *KCNT1* mutations, 4 previously studied *KCNT1* mutations, and one previously unpublished *KCNT1* variant of unknown significance. We analysed the properties of KCNT1 potassium currents and attempted to find a correlation between the changes in KCNT1 characteristics due to the mutations and severity of the neurological disorder they cause. *KCNT1* mutations identified in patients with epilepsy were introduced into the full length human *KCNT1* cDNA using quick-change site-directed mutagenesis protocol. Electrophysiological properties of different *KCNT1* constructs were investigated using a heterologous expression system (HEK293T cells) and patch clamping. All mutations studied, except T314A, increased the amplitude of KCNT1 currents, and some mutations shifted the voltage dependence of KCNT1 open probability, increasing the proportion of channels open at the resting membrane potential. The T314A mutation did not affect KCNT1 current amplitude but abolished its voltage dependence. We observed a positive correlation between the severity of the neurological disorder and the KCNT1 channel open probability at resting membrane potential. This suggests that gain of function *KCNT1* mutations cause epilepsy by increasing resting potassium conductance and suppressing the activity of inhibitory neurons. A reduction in action potential firing in inhibitory neurons due to excessively high resting potassium conductance leads to disinhibition of neural circuits, hyperexcitability and seizures.

## 1. Introduction

*KCNT1* encodes a sodium activated potassium channel, also known as SLACK (Sequence Like a Calcium Activated K^+^ channel), K_Ca_4.1 or Slo2.2 [1]. The gene is highly expressed in the nervous system, and the KCNT1 channel is thought to regulate neuronal excitability by modulating depolarization following repetitive firing of action potentials [1,2,3].

In 2012 we identified heterozygous *KCNT1* missense mutations in families and individuals with autosomal dominant nocturnal frontal lobe epilepsy (ADNFLE), now known as autosomal dominant sleep-related hypermotor epilepsy (ADSHE) [4]. The mutations were either inherited or occurred de novo and were associated with focal seizures often arising from sleep. Concurrently, Barcia et al. reported de novo *KCNT1* missense mutations in sporadic cases of the developmental and epileptic encephalopathy (DEE) syndrome known as epilepsy of infancy with migrating focal seizures (EIMFS) [5]. This type of infantile onset epilepsy is usually severe, with frequent focal seizures which are refractory to available epilepsy treatments. Bonardi et al. (2021) recently described 64 different *KCNT1* mutations identified in 248 individuals from around the world which provides insights into the clinical course and attempted management of KCNT1-related epilepsy [6].

To date, all but two reported *KCNT1* mutations in epilepsy are heterozygous missense mutations, predicted to cause a single amino acid change in the KCNT1 potassium channel. The exceptions are a homozygous missense mutation, c.G2896A, A966T, reported in an individual with the epileptic encephalopathy Ohtahara Syndrome [7] and a heterozygous in-frame deletion resulting in the deletion of a single amino acid, Gln550, causing EIMFS [8]. KCNT1-related epilepsy affects both children and adults. Affected individuals display a spectrum of epilepsy phenotypes, ranging from intermittent focal seizures, most often in the frontal lobe and with an onset in adolescence, to highly frequent seizures beginning in the first few months of life and involving multiple brain regions. KCNT1-related epilepsy can be associated with comorbidities including intellectual disability, autism, and behavioural features and can lead to premature death [6,9]. Explanations for the pathologies and different phenotypes seen with *KCNT1* mutations are not known and require investigation of the biological effects of the mutations and any differential effects. Thus far, the effects of a relatively small number of *KCNT1* mutations have been analysed by electrophysiology to look at their effects on KCNT1 channel properties and to date each mutation has led to increased potassium current [10,11].

In this study we investigated the effects of a large series of different disease causing *KCNT1* patient mutations on the electrophysiological properties of the KCNT1 channel, analysed in the same system and at the same time, to provide consistency in making intra-experiment comparisons. We analysed the properties and kinetics of the mutated KCNT1 channel, beyond K^+^ current amplitude, and looked for patterns between the observed effects and the phenotypes of the patient(s) with the particular mutation. The *KCNT1* mutations analysed in this study have all been identified in affected individuals and include nine not previously investigated by electrophysiology (T314A, N449S, L781V, E893K, M896V, F932L, S937G, L942F, and A965T) [6]. Four patient mutations that have previously been analysed (G288S, R398Q, R928C and R961H) were included for inter-experiment comparison [10]. One KCNT1 variant, S937G, identified in a sibling pair with severe epilepsy and co-morbidities, is previously unpublished and had been clinically classified as a variant of unknown significance. We have used electrophysiological analyses to investigate its effects on the KCNT1 channel to assess its mutational status.

## 2. Results

**Figure 1:**
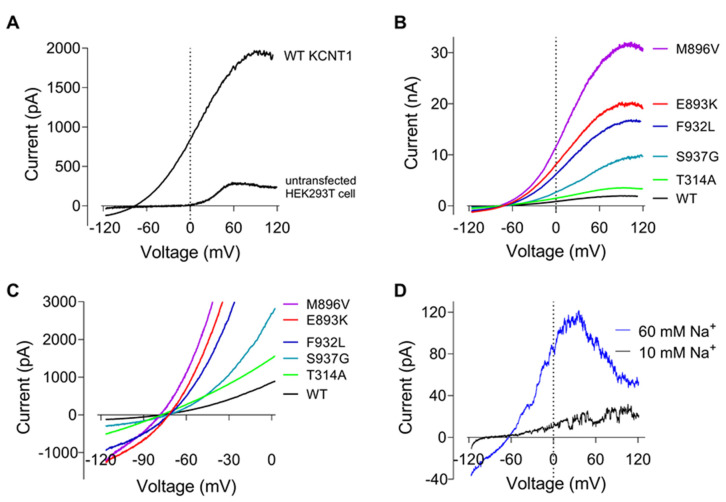
Current-voltage plots of WT and mutant KCNT1 channels expressed in HEK293T cells. (**A**) I-V plots recorded in response to 100 ms ramps ranging from −120 to 120 mV in a cell transfected with WT *KCNT1* and an untransfected cell. (**B**). Representative I-V plots of KCNT1 channels carrying GoF mutations. (**C**). The same data as on panel B, shown at a different scale. (**D**). WT KCNT1 currents recorded in inside-out patch in the presence of 10 mM or 60 mM intracellular Na^+^.

We selected 8 *KCNT1* mutations that we recently published in patients with *KCNT1*-related epilepsy that have not previously been functionally analysed: T314A, N449S, L781V, E893K, M896V, F932L, L942F and A965T [6], and one *KCNT1* variant of unknown significance, S937G, for our analyses. We also included 4 *KCNT1* mutations which have been previously functionally analysed by electrophysiology to allow for inter-experiment comparisons. To measure K^+^ currents mediated by KCNT1 channels we employed whole cell patch clamping of HEK293T cells transfected with plasmids containing cDNA encoding either wild type (WT) or mutant YFP-tagged KCNT1 protein. WT KCNT1 currents elicited by 100 ms voltage ramps ranging from −120 to 120 mV exhibited strong outward rectification between −120 and 40 mV and saturation at voltages above 50 mV (Figure 1A). All but one of the novel and previously described *KCNT1* mutations associated with epilepsy produced larger K^+^ currents, compared to WT, with I-V plots of similar shape (Figure 1B). The mutation that produced K^+^ current with an amplitude similar to that of WT KCNT1, T314A, also abolished outward rectification of the current without affecting current saturation at highly positive potentials (Figure 1B,C). Judging by the reversal potentials of the I-V plots, none of the mutations appreciably affected the selectivity of the channel pore (Figure 1C). I-V plots of WT KCNT1 current recorded in inside-out patches retained non-linear characteristics of whole-cell I-V plots (Figure 1D). Increasing Na^+^ concentration on the intracellular surface of the membrane from 10 mM to 60 mM resulted in a significant increase in current amplitude, as expected, but also in a bell-shaped I-V plot, suggesting voltage dependent block of the channel pore by intracellular Na^+^ at potentials above 40 mV. It is likely that whole-cell current saturation at highly positive membrane potentials accompanied by increased current noise is also a result of voltage-dependent block of the channels by Na^+^ (Figure 1A).

**Figure 2:**
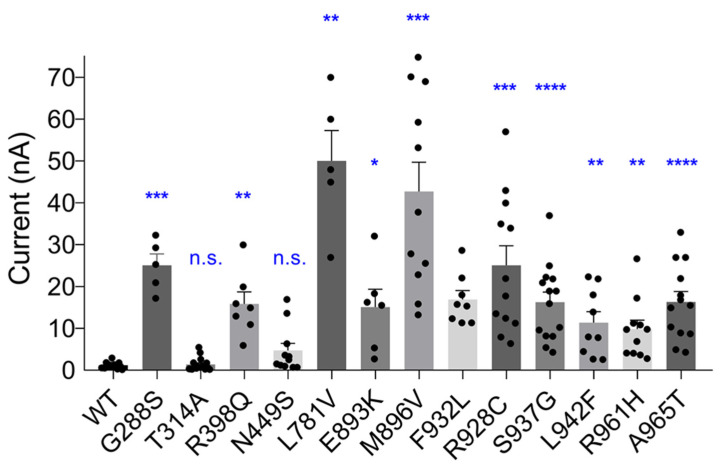
The effect of GoF mutations on KCNT1 current amplitude. The average amplitudes of WT and GoF mutant KCNT1 currents, measured at +10 mV using I-V plots similar to those shown in Figure 1. Brown-Forsythe and Welch’s ANOVA tests with multiple comparisons produced significant difference between the amplitude of WT and each mutant current, except T314A and N449S. Calculated individual *p* values were as follows: G228S—0.0009; T314A—0.6136; R398Q—0.0022; N449S—0.0535; L781V—0.0026; E893K—0.0224; M896V—0.0001; F932L—0.0001; R928C—0.0004; S937G—<0.0001; L942F—0.0049; R961A—0.0024; A965T—<0.0001. The dots on the graph represent current amplitudes in individual cells; the asterisks denote the level of the significance (*—<0.05; **—<0.01; ***—<0.001; ****—<0.0001); n.s.—not significant. (Note: The amplitudes of the largest currents are underestimated due to a residual uncompensated series resistance. See Section 4).

To compare the amplitudes of K^+^ currents mediated by WT and mutant KCNT1 channels, HEK293T cells were transfected with the same amounts of plasmids carrying cDNA of ether mutant or WT *KCNT1* and the measurements were performed within a short time window between 24 and 28 h post-transfection. Employment of *KCNT1* constructs tagged with YFP allowed selection of transfected cells with similar levels of fluorescence and therefore similar levels of KCNT1 protein expression. In the control experiments all YFP-tagged *KCNT1* constructs were confirmed to produce K^+^ currents indistinguishable from those of the corresponding untagged versions of *KCNT1*. As expected, epilepsy-causing mutations significantly increased the amplitude of KCNT1 currents (Figure 2). However, as stated above, there was a notable exception of the T314A mutation, which did not affect the amplitude of the current (*p* = 0.6136).

**Figure 3:**
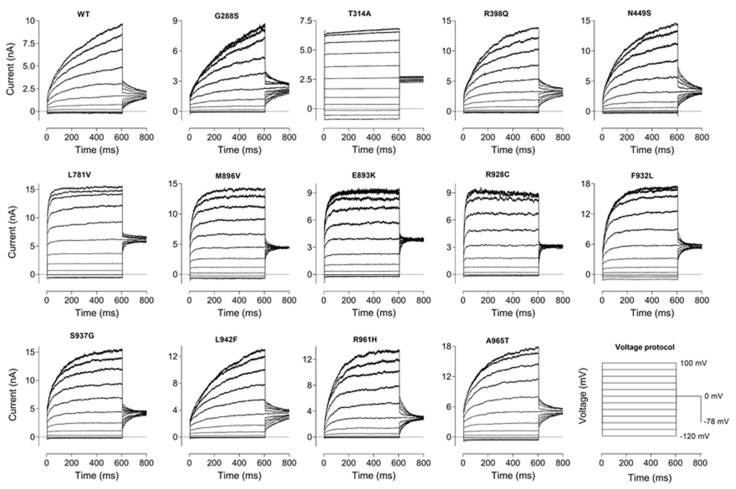
The effect of GoF mutations of the kinetics of KCNT1 currents. WT and Mutant KCNT1 currents were recorded in response to the voltage protocol shown in the lower right-hand corner.

**Figure 4:**
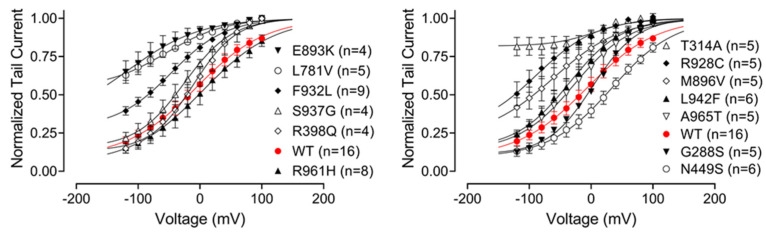
The effect of GoF mutations on the relative open probability of KCNT1 channels. Apparent *P*_o_ curves of WT and mutant KCNT1 channels were obtained from tail currents normalised to the instantaneous tail current amplitude corresponding to a voltage step to 100 mV using recordings similar to those shown in Figure 3. Each data set was fitted with the Boltzmann equation (Section 4, Equation (1)).

We observed that some of the mutations affected not only the amplitude of the current, but also the kinetics of activation (Figure 3), and the voltage dependence of the apparent open probability (Figure 4). Out of 13 mutations, seven (G288S, R398Q, N449S, S937G, L942F, R961H and A965T) had little effect on the time constant of KCNT1 current activation at 600 ms steps to 60–100 mV (τ = 100 ÷ 200 ms), five (L781V, E893K, M896V, R928C and F932L) significantly accelerated activation kinetics (τ = 20 ÷ 50 ms), and one (T314A) abolished activation altogether (Figure 3).

Using normalised tail currents, we have constructed apparent *P*_o_ curves which revealed a weak voltage dependence of KCNT1 open probability (Figure 4). The average slope of the apparent *P*_o_ curves (48 ± 2, *n* = 13) suggested a presence of a gating charge of about 0.5, however, the nature of this voltage dependence is not clear. Neither WT nor mutant KCNT1 channels were completely closed even at very negative potentials. Mutations that accelerated current activation at positive potentials also significantly increased minimum *P*_o_ at negative potentials, whereas T314A mutant exhibited virtually no voltage dependence at all (Figure 4).

The *KCNT1* variant c.2809A > G, p.S937G is previously unpublished and functionally uncharacterised. The heterozygous variant was identified in siblings affected with epilepsy syndromes and co-morbidities (See Supplementary Materials) previously associated with mutation of *KCNT1*. Due to not having been reported previously and the inability to determine the inheritance pattern of the mutation, it was classified as a variant of unknown significance (See Supplementary Materials for details of the mutation and the clinical phenotypes of the affected siblings). We have included the *KCNT1* S937G variant in this study to analyse its effects on the KCNT1 channel to assist in clinically classifying this variant. Our analysis showed that KCNT1 with this mutation produces a K^+^ current of significantly larger amplitude compared to WT KCNT1, but with the kinetics and apparent *P*_o_ similar to that of the normal channel (Figure 1, Figure 2, Figure 3 and Figure 4, Table 1).

**Figure 5:**
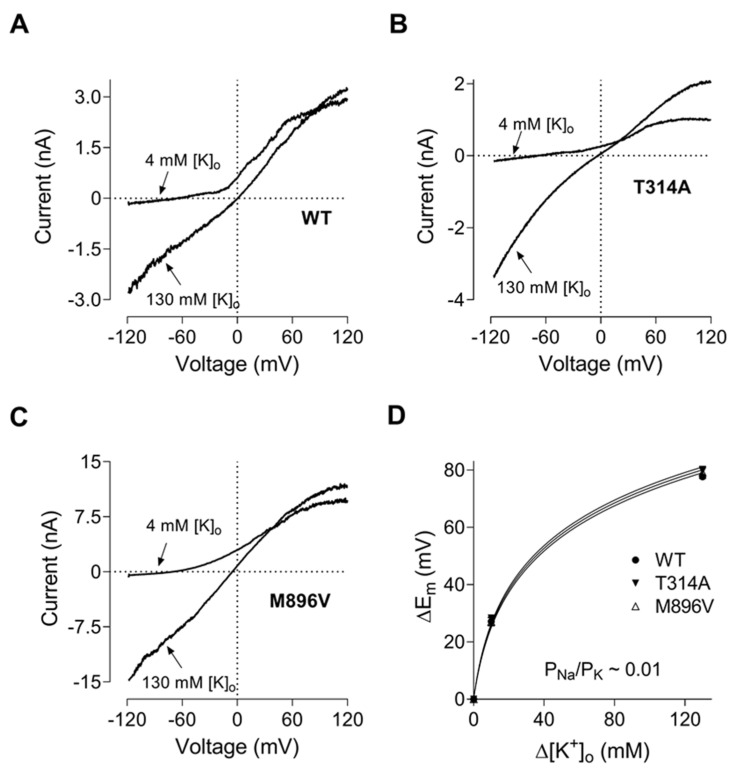
T314A and M896V GoF mutations do not alter KCNT1 pore permeability to Na^+^. (**A**). WT KCNT1. (**B**). T314A KCNT1. (**C**). M896V KCNT1. The I-V plots were recorded in response to 100 ms voltage ramps from −120 mV to 120 mV in the control bath solution (4 mM K^+^) and in a solution with 126 mM NaCl replaced with 126 mM KCl. (**D**). The shifts in the membrane potential caused by replacing NaCl with KCl were calculated using I-V plots similar to those shown in panels (**A**–**C**) and plotted against the KCl concentration changes. The solid curves are the fits of GHK equation (Equation (2), Section 4) to the experimental data.

To date it has been considered that the increased amplitude of the KCNT1 mediated current underlies the pathogenicity of *KCNT1* mutations. However, the T314A mutation investigated in this study produced a K^+^ current of a similar amplitude to the WT. The channel with the T314A mutation lacked the voltage dependence present in WT KCNT1 and other mutants (Figure 1, Figure 2, Figure 3 and Figure 4). This mutation was identified in a child with late onset (15 months) developmental and epileptic encephalopathy, with global and severe neurodevelopmental delay. Interestingly, T314A is the only *KCNT1* mutation reported to date located in the P-loop domain of the KCNT1 channel [6]. Considering that the *KCNT1* T314A mutation challenges the accepted paradigm of the pathobiology underlying KCNT1-epilepsy, we have investigated its properties in more detail. A crude estimation of the reversal potential using I-V plots (Figure 1) might not have been sufficient to ascertain the subtle changes in the KCNT1 selectivity for K^+^ over Na^+^ caused by mutations. Therefore, we investigated T314A selectivity, and compared it to the selectivity of WT and M896V KCNT1, by replacing different amounts of NaCl in the bath solution with KCl (Figure 5A–C) and calculating KCNT1 permeability for Na^+^ relative to K^+^ (P_Na_/P_K_) using the shifts in the reversal potentials of the I-V plots (Figure 5D) and modified GHK equation (Equation (2), Section 4). The data suggested that T314A mutation does not change the selectivity of KCNT1 and the relative permeability for Na^+^ remains at approximately 0.01 for WT, T314A and M896V KCNT1 channels.

**Figure 6:**
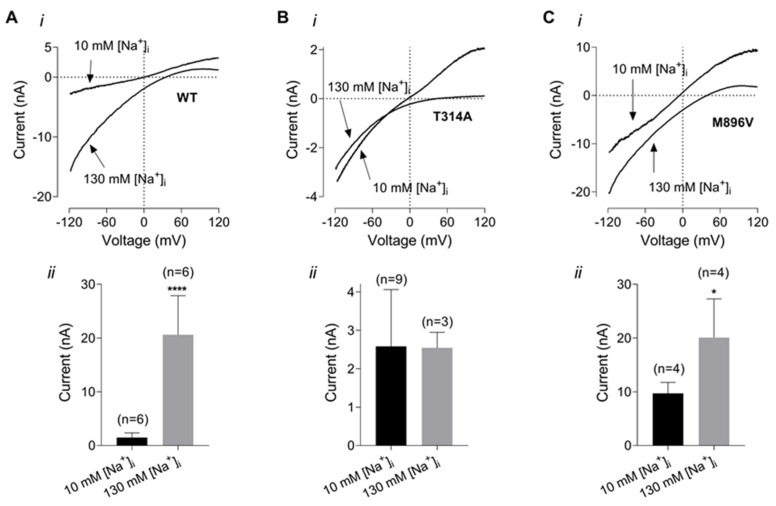
Dependence of WT and mutant KCNT1 currents on intracellular Na^+^ concentration. (**A**). WT KCNT1. (**B**). T314A KCNT1. (**C**). M896V KCNT1. The I-V plots (i) were recorded in response to 100 ms voltage ramps from −120 mV to 120 mV in the bath solution with 130 mM K^+^ and using either control pipette solution or pipette solution with 130 mM K^+^ replaced with 130 mM Na^+^. The amplitudes of the inward K^+^ currents recorded at −100 mV using low (10 mM) and high (130 mM) intracellular Na^+^ are compared on the panels below (ii). *—<0.05; ****—<0.0001.

Another possibility that could explain pathogenic effects of T314A mutations is a steeper, compared to WT, dependence on intracellular Na^+^. In the following experiment we have compared dependence of WT, T314A and M896V current amplitudes on intracellular Na^+^ concentration. The amplitudes of KCNT1 currents were measured at −100 mV in the presence of 130 mM K^+^ in the bath (126 mM NaCl in the control bath solution was replaced with 126 mM KCl) and using the pipette solution containing either 10 mM Na^+^ (control intracellular solution) or 130 mM Na^+^ (80 mM K glutamate and 40 mM KCl in the control intracellular solution were replaced with 80 mM Na glutamate and 40 mM NaCl, respectively) (Figure 6). As expected, increasing intracellular Na^+^ concentration from 10 mM to 130 mM potentiated WT KCNT1 current amplitude several fold (Figure 6A). In contrast, the amplitude of the T314A current did not change in response to increased intracellular Na^+^ concentration. For comparison, the amplitude of the M896V current did increase with higher intracellular Na^+^ concentration, however, this increase was significantly smaller than in WT KCNT1 current (about 2 fold vs. 14 fold).

A shift in KCNT1 amplitude dependence on intracellular Na^+^ towards lower concentrations has previously been reported for some KCNT1 mutants and has been suggested as an explanation of larger currents produced by these mutations at resting levels of intracellular Na^+^ [11]. Supporting this notion, at least for M896V mutant, the current amplitudes produced by WT and M896V mutant KCNT1 were vastly different in the presence of 10 mM Na^+^, but indistinguishable at saturating levels of intracellular Na^+^ (Figure 6A,C).

**Figure 7:**
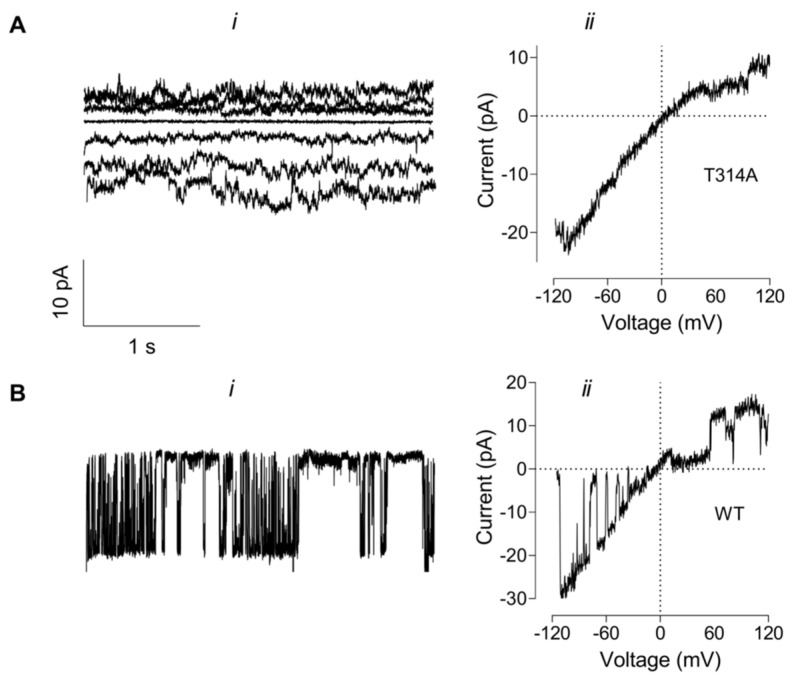
Membrane currents recorded in inside-out patches from the cells expressing T314A or WT KCNT1. (**A**). T314A KCNT1 current traces recorded in response to 3 s voltage steps ranging from −60 mV to 60 mV (i) or a voltage ramp from −120 mV to 120 mV (ii). (**B**). WT KCNT1 current trace recorded in response to 3 s voltage step to −40 mV (i) or a voltage ramp from −120 mV to 120 mV (ii). All traces were recorded using symmetrical K^+^ (130 mM) and Na^+^ (20 mM) concentrations in the bath and the pipette solutions.

Despite multiple attempts we were unable to record discernible single channel opening events in membrane patches expressing T314A KCNT1 (Figure 7A). The noisy current traces were consistent with the presence of several ion channels with a small conductance exhibiting inward rectification in symmetrical K^+^ concentration (130 mM) solutions (Figure 7A(i)). The instantaneous I-V plots obtained by applying a ramp voltage protocol to the inside-out patches were similar to the I-V plots obtained on the whole cells expressing T314A mutant channels (Figure 7A(ii) and Figure 6B). For comparison, inside-out patches of cells expressing other KCNT1 construct consistently produced single channel currents under the same recording conditions (Figure 7B, WT KCNT1 single channel recording is shown).

## 3. Discussion

In excitable cells, those that generate action potentials, the textbook roles of K^+^ channels are to control the resting membrane potential, regulate membrane resistance, and to control the repolarisation rate of action potentials and the action potential frequency [12]. In neuronal cells, in general, activation of K^+^ channels counteracts Na^+^- and Ca^2+^-mediated excitation. Not surprisingly, dysfunction of K^+^ channels can cause a multitude of neurological disorders [13,14]. Loss of function mutations in different types of K^+^ channels lead to increased excitability of neuronal circuits in the brain or spinal cord [13]. Unexpectedly, gain of function mutations in some types of K^+^ channels, including KCNT1 studied here, can also lead to hyperexcitability of neuronal circuits in the brain and consequently seizures [14,15]. A simple explanation of such a phenomenon is that these gain-of-function (GoF) mutant K^+^ channels are mainly expressed in inhibitory neurons and silencing of the inhibitory neurons due to abnormally large K^+^ conductance leads to disinhibition of the neuronal circuits and therefore increased excitability [14]. This notion is supported by the recent data obtained using a mouse model expressing human Y796H GoF *KCNT1*, demonstrating that increased Na-dependent K^+^ currents (Ik_Na_) impair GABAergic neuron excitability and alter synaptic connectivity [16]. Another recent study of a mouse model expressing L437F GoF *KCNT1* has revealed that KCNT1 channels are predominantly expressed in GABAergic parvalbumin-positive interneurons of the hippocampus, which exhibit a significantly reduced excitability, compared to this type of interneurons in the WT mice [17]. In contrast, data obtained from induced pluripotent stem cells (iPSC)-derived neurons expressing homozygous *KCNT1* P924L GoF mutation suggest the possibility of a different mechanism [18]. P924L-expressing neurons with Ik_Na_ increased several fold, responded to stimulation with a higher number of APs as well as with higher maximal firing rates, suggesting that *KCNT1* GoF mutations may increase excitability of the excitatory neurons [18]. The reason for such a discrepancy is not clear, however, this may indicate that different *KCNT1* GoF mutations cause epilepsy by different mechanisms.

Analysis of a *KCNT1* mutation classified as a variant of unknown significance (S937G), and therefore not reported clinically, has shown that it produces K^+^ currents with the characteristics similar to those of some other pathogenic *KCNT1* mutants investigated here (Table 1). Based on the electrophysiological effects of this variant it would now be classed as pathogenic. This highlights the importance of functional characterisation, and demonstrates the power of electrophysiological data, to assist in the classification of pathogenicity for ion channel variants, thus aiding in the genetic diagnoses and clinical care of patients.

**Figure 8:**
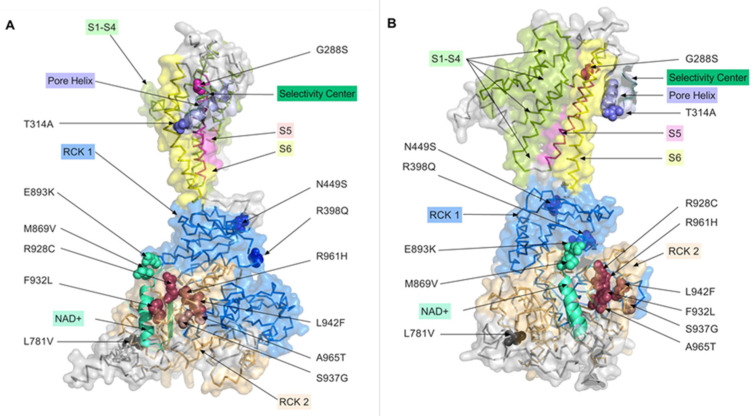
Distribution of the studied mutations on the 3D model of the KCNT1 channel. Mutations analysed in this paper were mapped onto the 3D structure of the chicken KCNT1 homolog, Slo2_2 in the open conformation deposited in the rcsb.org Protein Data Bank (PDB, http://doi.org/10.2210/pdb5U70/pdb, accessed on 3 November 2022) [20,21], with colours corresponding to the functional domains they are located in (see Section 4). The projection of a single KCNT1 subunit shown in panel (**B**) is obtained by anticlockwise rotation of the projection shown in panel (**A**) by 90 degrees around the vertical axis.

Seven of the 13 mutations studied in this work are located in, or adjacent to, the RCK2 domain, two are in the NAD binding domain, two in the RCK1 domain, one is in the P-loop and another one in the S5 domain (Table 1, Figure 8). The majority of known mutations that cause epilepsy are localised in the intracellular RCK domains and the membrane spanning S5 domain [6]. Many of the epilepsy causing mutations increase the sensitivity of KCNT1 channel to intracellular Na^+^, which explains large macroscopic K^+^ currents mediated by these mutant KCNT1 channels at the resting levels of Na^+^ concentration [11]. However, there is evidence that epilepsy-causing mutations increase positive cooperativity of the KCNT1 channel opening, which can also explain the increase of the macroscopic current amplitude [19].

The only mutation found so far within the pore-forming P-loop domain is T314A (Figure 8). Close proximity of this mutation to the selectivity centre explains drastically reduced single channel conductance of T314A mutant and the reduced, compared to the other epilepsy-causing mutations, whole-cell K^+^ conductance. All mutations investigated in this study, except for T314A, produced KCNT1 currents of larger amplitude, compared to the WT channel. Some mutations have also affected the kinetics of activation as well as the voltage dependence of the apparent open probability. To gain insights into the relation between the characteristics of the mutant KCNT1 channel with the severity of the epilepsy caused by the mutations in humans, we employed Spearman correlation analysis. Using the available clinical data, each mutation was assigned the score between 1 (less severe) and 3 (more severe) (Table 1) and Spearman correlation coefficient, r, between the severity of the disease, KCNT1 current amplitude, relative *P*_o_ at −80 mV, and kinetics was calculated using GraphPad Prism 8 (GraphPad Software Inc., San Diego, CA, USA). The analysis suggested that there is no correlation between the severity of disease and KCNT1 current amplitude (r = 0.29; *p* = 0.336). There was, however, a strong correlation between the severity and the apparent *P*_o_ around resting membrane potential (r = 0.72; *p* = 0.007). There were also correlations between the disease severity and KCNT1 kinetics (r = −0.70; *p* = 0.010), most likely due to a very strong correlation between the current kinetics and the *P*_o_ (r = −0.82; *p* = 0.003). This suggests that for the mutations tested here, open probability of KCNT1 channels at the resting potential is a strong predictor of the severity of the disease. This also suggests that the most likely mechanism, by which these KCNT1 mutant channels affect neuronal excitability, is by increasing resting membrane K^+^ conductance and therefore working as a brake for action potential firing. In such a case the effects of *KCNT1* mutations on the neuronal excitability must be more pronounced in the inhibitory than the excitatory neurons. This corresponds well with the data obtained from mouse models of KCNT1-mediated epilepsy [16,17]. Importantly, the effects of T314A, which abolished voltage and Na^+^ dependence (at least between 10 and 130 mM) of KCNT1, suggest that the pathogenicity of this mutation is due to increased resting K^+^ conductance. Increased resting K^+^ conductance acts as a break for action potential firing, which strongly indicates the predominant effect of *KCNT1* GoF mutations on the inhibitory neurons [22,23]. Similarly, the pathogenicity of the gain-of-function mutations in Kv7.2 and Kv7.3 K^+^ channels found in patients with epileptic encephalopathies, which increase the open probability of the channels at the resting membrane potential, was also suggested to be due to a decreased excitability of the inhibitory neurons and disinhibition of the neuronal circuits [24].

Clearly, GoF mutations which do not affect the *P*_o_ of the KCNT1 channel at the resting membrane potential also increase the resting K^+^ conductance due to the fact that KCNT1 does not completely close even at very negative potentials. To ascertain whether the KCNT1 current amplitude can be used as a predictor of the disease severity for these mutations, the mutations that increase KCNT1 apparent *P*_o_ at negative potentials were removed from consideration. In this case, the analysis revealed that, indeed, *KCNT1* mutations that have voltage dependence similar to WT KCNT1 do exhibit strong correlation between the current amplitude and the disease severity (r = 0.72, *p* = 0.033).

It should be acknowledged, however, that the scoring of the mutations’ phenotypic severity has its limitations due to variations in availability of clinical data, different numbers of patients carrying specific mutations, and possible co-morbidities unrelated to *KCNT1* mutations. The epilepsy phenotypes associated with each of the *KCNT1* mutations could be expanded in the future, as informed by further genetics studies, which may alter the scoring of the mutation phenotypes.

In conclusion, we investigated the effects of nine novel and four known *KCNT1* mutations on the biophysical properties of KCNT1 currents, including whole cell current amplitude, apparent open probability, current kinetics, and single channel conductance (T314A mutant). The only difference that we found between WT KCNT1 and T314A that could be described as gain of function is the open probability at the resting potential, which is significantly higher in T314A. The analysis of correlation between the symptoms caused by the different *KCNT1* mutations and the properties of KCNT1 current suggests for the first time, that KCNT1 *P*_o_ at resting membrane potential is a strong predictor of the severity of KCNT1-related epilepsy and supports the notion that pathogenicity of *KCNT1* mutations is caused by their effects on the resting K^+^ membrane conductance, rather than the current amplitude at positive potentials.

## 4. Materials and Methods

### 4.1. Patient Mutations Analysed

Patients provided informed consent. This study was approved by the IRB of UniSA (Protocol: 0000032998 of 29 August 2016). This study includes one previously unpublished KCNT1 variant, c.2809A > G, p.S937G, identified by whole exome sequencing analysis of previously unreported siblings with epilepsy. The mother is negative for the *KCNT1* S937G variant and the father is unavailable for genetic analysis. The male sibling had Early Infantile Epileptic Encephalopathy (EIEE), evolving to West Syndrome (WS) and a Lennox Gastaut Syndrome (LGS) phenotype with severe intellectual disability, quadriplegia, delayed myelination, dyskinesias, feeding difficulties and microcephaly. The female sibling had a milder epilepsy phenotype than her brother, with infantile onset epilepsy evolving to refractory autosomal dominant sleep-related hypermotor epilepsy (ADSHE), severe intellectual disability, quadriplegia, dyskinesias, and non-specific white matter change on MRI (See Supplementary Materials for more information).

### 4.2. Cell Culture and Transfections

Human embryonic kidney 293T cells (HEK 293T, American Type Culture collection, Rockville, MD, USA) were maintained at 37 °C, 5% CO_2_ atmosphere, in Dulbecco’s modified Eagle’s medium (ThermoFisher, Australia) supplemented with 10% (*v/v*) of fetal calf serum (Gibco, New York, NY, USA), 2 mM L-glutamine, and 1% (*v/v*) non-essential amino acids. To express *KCNT1* constructs HEK293T cells plated on glass cover slips were transfected using Attractene Transfection Reagent (Qiagen, Germany) according to the manufacturer’s instructions.

### 4.3. Mutagenesis

The full-length human *KCNT1* cDNA in pCMV-Entry vector (Cat#SC311132) was sourced from Origene, Rockville, MD, USA. The mutagenesis of *KCNT1* cDNA was done using Quickchange lightning site-directed mutagenesis kit from Agilent Technologies Inc., Santa Clara, CA, USA (Cat# 210518) to introduce following mutation: G288S (Gly288Ser), T314A (Thr314Ala), R398Q (Arg398Gln), N449S (Asn449Ser), L781V (Leu718Val), E893K (Glu893lys), M896V (Met896Val), L924F (Leu942Phe), R928C (Arg928Cys), F932L (Phe932Leu), S937G (Ser937Gly), R961H (Arg961His) and A965T (Ala965Thr). Primers were designed to introduce specific mutations (www.agilent.com/genomics/qcpd (accessed on 20 April 2018)), and PCR amplification was done using high-fidelity DNA polymerase. Following PCR, Dpn1 endonuclease treatment was performed to digest template DNA, leaving the nicked PCR product with desired mutation. The digested PCR product with EcoR1 and Xho1 restriction enzymes was ligated with pCMV-entry vector using T4-Ligase (NEB# M0202S) and then transformed in to XL10-Gold ultra-competent *E. coli* cells (Agilent Technologies # 210518). Mutated sequences were verified by Sanger sequencing. In addition, we have generated YFP-6His tagged versions of wild type and mutant KCNT1 using 3 piece ligation (6His-YFP, KCNT1, pCMV-entry vector).

### 4.4. Patch Clamping

Whole-cell patch-clamp recording of KCNT1 mediated current was performed using transiently transfected HEK293T cells 18–28 h post transfection. The transfected cells were identified using Olympus IMT2 microscope and the fluorescence of the YFP tag present in each *KCNT1* construct. Cells exhibiting fluorescent intracellular inclusions and structural abnormalities, as well as the cells that were too bright or too dim were excluded from the analysis. Whole-cell patch clamping was performed at room temperature (23 °C) using a computer based patch-clamp amplifier (EPC-9, HEKA Elektronik) and PULSE software (HEKA Elektronik). The control bath solution contained 140 mM NaCl, 4 mM KCl, 2 mM CaCl_2_, 2 mM MgCl_2_ and 10 mM HEPES adjusted to pH 7.4 with NaOH. The control internal solution contained 80 mM K gluconate, 50 mM KCl, 10 mM NaCl, 1mM MgATP, 10 mM EGTA and 10 mM HEPES adjusted to pH 7.3 with KOH. Patch pipettes were pulled from borosilicate glass and fire polished to give a pipette resistance between 1 and 2 MΩ. Series resistance ranged between 2.5–4 MΩ and was 80–90% compensated. In the experiments aimed at comparing KCNT1 current amplitudes the plasmids containing WT or mutant *KCNT1* cDNA were transfected into HEK293T cells at the same amounts (0.8 µM in 35 mm Petri dish) and cells were used for patch clamping within a short time window 24–28 h post transfection. This resulted in some mutant KCNT1 currents of very large amplitudes, and a significant voltage error due to the residual uncompensated series resistance. Even with 90% compensation the residual series resistance of 0.3 MΩ could in extreme cases cause a 25–30% voltage error. These cells were still included in the data presented in Figure 2, and it should be kept in mind that the currents with the amplitude above 25 nA are underestimated. However, underestimation of the amplitude of the very large currents in Figure 2 does not affect the conclusions of this paper. For the experiments aimed at comparing relative open probability of different KCNT1 mutant channels only cells with the voltage error less than 10% due to the residual series resistance were used for the analysis. For some KCNT1 mutants the amount of cDNA in the transfection mixture or the time post transfection were reduced to achieve expression of relatively smaller currents that could be properly voltage clamped.

### 4.5. Data Analysis

To obtain apparent (relative) open probability (*P*_o_) curves of KCNT1 channels, instantaneous tail currents recorded in response to voltage steps to 0 mV after test pulses between −120 and 100 mV, applied every 10 s in 20 mV increments, were normalised to the amplitude of the instantaneous tail current recorded after test pulse to 100 mV and plotted against corresponding test pulse voltage. The data points were fitted with the Boltzmann distribution with an offset of the form:

(1) $$P_{o}\left(V\right)=P_{\min}+\frac{1-P_{\min}}{1+e^{\left(\frac{V_{0.5}-V}{k}\right)}}\;$$

where *P*_min_ is an offset, *V* is the membrane potential, *V*_0.5_ is the half-maximal activation potential (*V*_0.5_ corresponds to the inflexion point of the *P*_o_ curve) and *k* is the slope factor.

The permeability of Na^+^ relative to K^+^ has been determined by replacing 10 mM and 126 mM of NaCl in the bath solution with the corresponding amounts of KCl and calculating the shifts in the reversal potentials of KCNT1 currents. The reversal potential shift data have been fitted with a modified Goldman-Hodgkin-Katz equation of the form:

(2) $$Y=58\log\frac{\left(4+X\right)+\left(145-X\right)A}{4+145A}\;$$

where *Y* is the shift in the reversal potential, *X* is the concentration of NaCl replaced with KCl, *A* is the relative permeability ratio (P_Na_/P_K_), 4 and 145 are concentrations of K^+^ and Na^+^ in mM, correspondingly, in the control bath solution.

### 4.6. 3D Modelling of KCNT1 Mutation Locations

The 13 mutations analysed in this paper were mapped to the 3-dimensional (3D) structure of the chicken KCNT1 homologue, SLO2_2, present in the Research Collaboratory for Structural Bioinformatics (RCSB) Protein Data Base (PDB) [25] with PDB ID: 5U70, which is the open conformation derived from cryo-EM analysis (PDB, http://doi.org/10.2210/pdb5U70/pdb, accessed on 3 November 2022) [20,21]. PDB file 5U70 was loaded into the PyMOL Molecular Graphics System, Version 2.0 Schrödinger, LLC, NY. Domains were annotated according to Hite et al. (2015) [20] and colour coded. The Pore Helix, Selectivity Centre and NAD+ binding domain are shown as cartoon renders of their structural features (e.g., alpha-helices) using coordinates adapted from Hite et al. (2015) [21] and Bonardi et al. (2021) [6]. Mutations analysed in this paper were mapped onto the 3D structure with colours corresponding to the functional domains they are located in. 3D images rendered in PyMOL were exported to PRISM 9 for Mac (GraphPad Software LLC San Diego, CA, USA) for labelling and publication.

## Figures and Tables

**Table 1:** Correlation of the effects of *KCNT1* mutations on the electrophysiological properties of KCNT1 currents with patient phenotypes. KCNT1 current amplitude is expressed as fold increase, compared to the amplitude of the WT KCNT1 current (derived from the data on Figure 2). Apparent *P*_o_ at −80 mV is taken from the data shown in Figure 4. The kinetics of KCNT1 current is expressed as a time constant of an exponential function fitted to KCNT1 current traces obtained in response to 600 ms voltage steps to 80 mV.

*KCNT1* Mutation/ Location	Patient Phenotype (Severity Score)	K^+^ Current Amplitude (Fold Increase)	Apparent P_o_ at −80 mV (%) (*n* = 4–9)	Kinetics τ (ms) (*n* = 4–8)
G288S /S5	Severe (EIMFS), less severe (2) (SHE) or unaffected	21	20 ± 2.8	197 ± 16
T314A /P-loop	Severe (atypical DEE with (3) severe ID)	1.2	82 ± 5.7	N/A
R398Q /RCK1	Severe (EIMFS or DEE), less (2) severe (SHE) or unaffected	13	25 ± 4.6	161 ± 11
N449S /RCK1	Less severe (1) (SHE with mild ID)	4	18 ± 3.5	175 ± 12
L781V /adj RCK2	Severe (3) (DEE and severe ID)	42	73 ± 2.6	33 ± 5
E893K /NAD+	Severe (3) (EIMFS)	13	76 ± 7.7	42 ± 5
M896V /NAD+	Severe (EIMFS or DEE) (2) or less severe (SHE)	36	54 ± 8.0	51 ± 4
R928C /RCK2	Less severe (1) (SHE or TLE)	21	67 ± 6.8	16 ± 3
F932L /RCK2	Moderate (2) (SHE with moderate ID)	14	52 ± 3.1	52 ± 3
S937G /RCK2	Moderate (2) (DEE or SHE)	14	33 ± 5.6	113 ± 6
L942F /RCK2	Less severe (1) (SHE)	10	39 ± 3.8	226 ± 9
R961H /RCK2	Moderate (SHE with (2) cognitive regression)	8	23 ± 4.7	88 ± 6
A965T /RCK2	Less severe (1) (SHE)	14	35 ± 6.3	139 ± 7

Abbreviations in the table: EIMFS—Epilepsy of Infancy with Migrating Focal Seizures; SHE—Sleep-Related Hypermotor Epilepsy; DEE—Developmental and Epileptic Encephalopathies; TLE—Temporal Lobe Epilepsy; ID—Intellectual Disability.

## Data Availability

Data supporting reported results can be requested from the corresponding author.

## Supplementary Materials

The following supporting information can be downloaded at: https://www.mdpi.com/article/10.3390/ijms232315133/s1.
