# Supplementary material for: Functional Effects of Epilepsy Associated KCNT1 Mutations Suggest Pathogenesis via Aberrant Inhibitory Neuronal Activity
Source: Int J Mol Sci. 2022 Dec 1;23(23):15133. doi: 10.3390/ijms232315133 (PMC9740882; doi:10.3390/ijms232315133)
Supplement: Supplementary file 1 [file ijms-23-15133-s001.zip › ijms-2043886-supplementary.pdf]

Supplementary data

The *KCNT1* variant c.2809A>G, p. S937G was identified by whole exome sequencing analysis of a male child with severe epilepsy and comorbidities. Sanger sequencing confirmed the presence of the c.2809A>G sequence change in the patient and showed that his sister, also affected with epilepsy and co-morbidities, had the variant, while their mother (unaffected) did not (data not shown). The father was not available for genetic testing, so the mode of inheritance of the variant was not able to be determined. The *KCNT1* variant has not been previously described in patients with *KCNT1*-related disorders and has also not been described as a known benign sequence change in the KCNT1 gene. *In silico* pathogenicity prediction tools (SIFT, PolyPhen2, Align GVGD and MutationTaster) provided contradictory results for the S937G substitution. It was classified as a variant of unknown significance. The boy is now 17 years old and had neonatal onset seizures with early infantile epileptic encephalopathy (i.e. with burst suppression) which evolved to West syndrome with infantile spasms, and now has a Lennox Gastaut Syndrome with daily seizures, which include generalized tonic clonic seizures, tonic seizures, absence seizures and myoclonic seizures. He has been treated with multiple anti-seizure medications (vigabatrin, valproate, zonisamide and topiramate) as well as the ketogenic diet, and his epilepsy is medically refractory. He is currently treated with clobazam, levetiracetam and lacosamide. He has global developmental delay, movement disorder with dyskinesias, feeding difficulties and microcephaly. Magnetic resonance imaging (MRI) of the brain has shown delayed myelination and diffuse mild volume loss affecting the cerebrum and cerebellum. His sister is now 16 years and also had infantile onset epilepsy. Her epilepsy is medically refractory and multiple anti-seizure medications have been tried, including carbamazepine, phenytoin, phenobarbital, topiramate, levetiracetam and valproic acid. She is now treated on the ketogenic diet, lamotrigine and lacosamide. Her phenotype is most consistent with sleep-related hypermotor epilepsy and she experiences nocturnal bilateral tonic-clonic seizures on a monthly basis. She also has a movement disorder with dyskinesias and ataxia. She is globally delayed. Brain MRI has shown non-specific white matter changes with mild to moderate subcortical and periventricular white matter volume loss. The clinical phenotypes of both siblings are consistent with those previously reported in patients with *KCNT1*-epilepsy (Bonardi et al 2021).
